# Mental health status and related influencing factors of COVID‐19 survivors in Wuhan, China

**DOI:** 10.1002/ctm2.52

**Published:** 2020-06-05

**Authors:** Chaomin Wu, Xianglin Hu, Jianxin Song, Dong Yang, Jie Xu, Kebin Cheng, Dechang Chen, Ming Zhong, Jinjun Jiang, Weining Xiong, Ke Lang, Yan Tao, Xiaoqin Lin, Guohua Shi, Liwen Lu, Longci Pan, Lei Xu, Xin Zhou, Yuanlin Song, Ming Wei, Junhua Zheng, Chunling Du

**Affiliations:** ^1^ Department of Pulmonary Medicine QingPu Branch of Zhongshan Hospital Affiliated to Fudan University Shanghai China; ^2^ Department of Pulmonary Medicine Zhongshan Hospital, Fudan University Shanghai China; ^3^ Department of Infectious Diseases Tongji Hospital Tongji Medical College Huazhong University of Science and Technology Wuhan China; ^4^ Department of Infectious Diseases Fengxian Guhua Hospital Shanghai China; ^5^ Department of Respiratory and Critical Care Medicine Shanghai Pulmonary Hospital Tongji University School of Medicine Shanghai China; ^6^ Medical College of Soochow University Suzhou China; ^7^ Department of Critical Care Medicine Ruijin Hospital Shanghai Jiao Tong University School of Medicine Shanghai China; ^8^ Department of Critical Care Medicine Zhongshan Hospital Fudan University Shanghai China; ^9^ Department of Respiratory Medicine Shanghai Ninth People's Hospital Shanghai Jiaotong University School of Medicine Shanghai China; ^10^ Department of Nursing Shanghai Pudong New Area Pulmonary Hospital Shanghai China; ^11^ Department of Pulmonary Medicine Qingpu Traditional Chinese Medicine Hospital Shanghai China; ^12^ Department of Pulmonary Medicine Fengxian Central Hospital Shanghai China; ^13^ Department of Traditional Chinese Medicine QingPu Branch of Zhongshan Hospital Affiliated to Fudan University Shanghai China; ^14^ Department of Emergency Medicine Gongli Hospital Shanghai China; ^15^ Department of Pulmonary Medicine Shanghai General Hospital School of Medicine in Shanghai Jiao Tong University Shanghai China; ^16^ Tuberculosis and Respiratory Department Wuhan Jinyintan Hospital Wuhan China; ^17^ Department of Urology Shanghai General Hospital School of Medicine in Shanghai Jiao Tong University Shanghai China

**Keywords:** COVID‐19, follow‐up, mental health, survivors

Dear editor,

In late December 2019, a novel contagious pneumonia named coronavirus disease 2019 (COVID‐19) has broken out in Wuhan, China.^1^ On January 30, 2020, World Health Organization (WHO) declared COVID‐19 as a Public Health Emergency of International Concern. On March 11, 2020, WHO characterized COVID‐19 as a pandemic.^2^, ^3^ Much research work has been done for hospitalized COVID‐19 patients, mainly in clinical characteristics.^4^ However, few studies have reported the post‐discharge follow‐up status, especially the mental health status of COVID‐19 survivors. Therefore, in this descriptive case series, we enrolled a large number of COVID‐19 survivors in Wuhan, China. We aimed to report the post‐discharge mental health status of these survivors and explore relevant influencing factors.

This study was conducted in Wuhan Jinyintan Hospital. All patients were confirmedly diagnosed with COVID‐19.^1^ The flowchart is shown in Figure S1. Eventually, 370 COVID‐19 survivors were included in this study. Verbal consent of follow‐up was obtained in all the 370 survivors. Survivors’ readmission status and the reasons were inquired. Post‐discharge respiratory symptoms were inquired. Whether the survivors worried about COVID‐19 recurrence was inquired. Whether the survivors worried about COVID‐19 infection to others (family members) was inquired. Home quarantine lifestyles status was inquired. Anxiety was measured using The Generalized Anxiety Disorder Screener (GAD‐7). Total score 0‐4 refers to no anxiety; total score 5‐21 refers to anxiety.^5^ Depression was measured using Patient Health Questionnaire‐9 (PHQ‐9). Total score 0‐4 refers to no depression; total score 5‐27 refers to depression.^6^

Statistical analysis was performed using SPSS (Version 24.0). Continuous variables were presented by mean ± standard deviation (SD) or median with inter quartiles (IQR). Categorical variables were presented by number with percentage. Student's *t*‐test and Chi‐square test were used as appropriate. *P* < .05 was statistically significant.

Clinical data and post‐discharge status were summarized in Table 1. The median time from discharge to follow‐up were 22 days (IQR 20‐30 days). Six (1.6%) survivors were readmitted to hospital during the follow‐up, including two for cough without SARS‐CoV‐2 RNA positive, two for pneumonia without SARS‐CoV‐2 RNA positive, one for transient SARS‐CoV‐2 RNA positive without pneumonia, and one for lumbar disease. No SARS‐CoV‐2‐positive pneumonia recurred in any survivors during the follow‐up.

**Table 1:** Clinical characteristics and post‐discharge status of the enrolled survivors (N = 370)

Parameters	All patients
Age (years)	50.5 ± 13.1
Male	203 (54.9%)
Huanan seafood market exposure	113 (30.5%)
Infection with family members	25 (6.8%)
Infected medical staffs	33 (8.9%)
Current smoking	21 (5.7%)
Common comorbidity	
Hypertension	79 (21.4%)
Diabetes	31 (8.4%)
Common symptoms and signs at disease onset	
Fever	326 (88.1%)
Highest temperature (°C)	38.7 ± 0.65
Cough	288 (77.8%)
Breathlessness or dyspnea	125 (33.8%)
Sputum	111 (30.0%)
Timeline	
Days from disease onset to admission	10 (7∼13)
Days from admission to discharge	12 (9∼14)
Days from discharge to follow‐up	22 (20∼30)
Post‐discharge status	
Readmission	6 (1.6%)
Readmission for cough without SARS‐CoV‐2 RNA positive	2
Readmission for pneumonia without SARS‐CoV‐2 RNA positive	2
Readmission for transient SARS‐CoV‐2 RNA positive without pneumonia	1
Readmission for lumbar disease	1
Readmission for recurrent SARS‐CoV‐2 pneumonia	0
Respiratory symptoms in post‐discharge period	
Cough	60 (16.2%)
Sputum	20 (5.4%)
Breathlessness after activity	45 (12.2%)
Worry about recurrence	173 (46.8%)
Worry about infection to others	174 (47.0%)
Both worry about recurrence and infection to others	136 (36.8%)
Home quarantine lifestyle	293 (79.2%)
Anxiety (GAD‐7 measurement)	50 (13.5%)
Depression (PHQ‐9 measurement)	40 (10.8%)
Comorbid anxiety and depression	23 (6.2%)
Willingness to return to hospital for health examination	356 (96.2%)

GAD‐7, The Generalized Anxiety Disorder Screener; PHQ‐9, Patient Health Questionnaire‐9.

Sixty (16.2%) survivors had post‐discharge cough and 45 (12.2%) had breathlessness after activity. Twenty (5.4%) survivors had sputum production during the follow‐up. One hundred seventy‐three (46.8%) survivors worried about recurrence and 174 (47.0%) worried about infection to others. Two hundred ninety‐three (79.2%) survivors took a home quarantine lifestyle. Fifty (13.5%) survivors occurred anxiety. Forty (10.8%) survivors occurred depression.

As shown in Table S1, survivors (39.2%) were most bothered by feeling nervous, anxious, or on edge. As shown in Table S2, a high proportion of 29.5% survivors were bothered by sleeping disorders. Four survivors (1.1%) once had thoughts of suicide in several days.

As shown in Table 2, survivors with post‐discharge respiratory symptoms, worry about recurrence, or worry about infection to others had significantly increased incidence of anxiety (*P* < .05). Female, or survivors with post‐discharge respiratory symptoms, worry about recurrence, worry about infection to others, or home quarantine lifestyle had significantly increased incidence of depression (*P* < .05). Anxiety and depression were not associated with age, family infection, comorbidity, and so on.

**Table 2:** Factors associated with anxiety or depression of the survivors (N = 370)

	With anxiety	Without anxiety		With depression	Without depression	
Variable	(n = 50)	(n = 320)	*P*‐value^a^	(n = 40)	(n = 330)	*P*‐value^b^
Age	52.9 ± 13.3	50.1 ± 13.1	.171	54 ± 14.2	50.1 ± 13.0	.074
Female	26 (52.0%)	141 (44.1%)	.294	24 (60.0%)	143 (43.3%)	.045*
Infection with family members	1 (2.0%)	24 (7.5%)	.255	2 (5.0%)	23 (7.0%)	.892
Infected medical staffs	3 (6.0%)	30 (9.4%)	.609	4 (10.0%)	29 (8.8%)	1.000
Current smoking	2 (4.0%)	19 (5.9%)	.824	1 (2.5%)	20 (6.1%)	.577
Common comorbidity						
Hypertension	13 (26.0%)	66 (20.6%)	.388	13 (32.5%)	66 (20.0%)	.068
Diabetes	2 (4.0%)	29 (9.1%)	.354	3 (7.5%)	28 (8.5%)	1.000
Respiratory symptoms in post‐discharge period						
Cough	15 (30.0%)	45 (14.1%)	.004*	17 (42.5%)	43 (13.0%)	<.001*
Sputum	7 (14.0%)	13 (4.1%)	.011*	9 (22.5%)	11 (3.3%)	<.001*
Breathlessness after activity	14 (28.0%)	31 (9.7%)	<.001*	15 (37.5%)	30 (9.1%)	<.001*
Worry about recurrence	34 (68.0%)	139 (43.3%)	.001*	32 (80.0%)	141 (42.7%)	<.001*
Worry about infection to others	37 (74.0%)	137 (42.8%)	<.001*	34 (85.0%)	140 (42.4%)	<.001*
Home quarantine lifestyle	44 (88.0%)	249 (77.8%)	.099	39 (97.5%)	254 (77.0%)	.003*

a *P*‐value: with anxiety versus without anxiety.

b *P*‐value: with depression versus without depression.

In this study, we conducted a post‐discharge follow‐up of COVID‐19 survivors. No SARS‐CoV‐2‐positive pneumonia was recurrent in this population during the follow‐up period. We identified one survivor with transient SARS‐CoV‐2 RNA turning into positive. However, the positive SARS‐CoV‐2 RNA soon turned into negative again (interval: 5 days) just when he was readmitted. We Chinese experts pointed out that SARS‐CoV‐2 RNA turning into positive in survivors is not equal to recurrence or re‐infection.^7^ There might be two reasons for transient SARS‐CoV‐2 RNA positive in survivors: first, it comes from the nucleic acid fragments of the inactivated SARS‐CoV‐2; second, the virus titer lowers to a level that can hardly be detected at discharge, the residual virus fluctuated at post‐discharge but would be soon cleared by body immunity. COVID‐19 survivors should not be overly worried for a rare event of recurrence, as we found a high proportion of survivors (46.8%) worried about recurrence.

An epidemic disease, such as SARS in 2003, generally accompanies with multiple psychiatric morbidities, including anxiety, depression, and even suicide.^8^ In our study, we found anxiety and depression existed in approximately 10% of COVID‐19 survivors. We also found a high proportion of 29.5% survivors were bothered by sleeping disorders. For those survivors with severe sleeping disorders, some medications could be prescribed to help them improve the sleep. Survivors with suicidality (1.1%) must be closely followed up and cared by psychiatrists.

We found anxiety and depression are significantly associated with post‐discharge residual symptoms, worry about recurrence, and worry about infection to others. Besides, females were more susceptible to depression. We clinicians should explain to survivors that residual respiratory symptom is common in the recovery period of pneumonia. As time goes by, most residual respiratory symptom would gradually disappear.

In Chinese national diagnosis and treatment scheme of COVID‐19,^9^ all COVID‐19 survivors are suggested to take a post‐discharge home quarantine lifestyle for 2 weeks. The main requirements of home quarantine lifestyle included living in single drafty room, reduction of close contact with family, separate meals, and avoidance of outdoor activity. This conduct is necessary to avoid unexpected infections to others. However, we found home quarantine lifestyle is associated with increased incidence of depression. Therefore, effective measures need to be taken to relieve the depression caused by home quarantine lifestyle, such as online chat or video chat with family, indoor exercise, and so on.

In summary, about 10% of COVID‐19 survivors develop anxiety or depression, because of post‐discharge residual respiratory symptoms, worry about recurrence, and infection to others. Female COVID‐19 survivors are more susceptible to depression. COVID‐19 survivors should not be overly worried about a rare event of recurrence. In addition, depression caused by home quarantine lifestyle should also be noted and relieved.

## CONFLICT OF INTEREST

The authors declared no conflict of interest.

## Supporting information

Supporting information

Supporting information

## Data Availability

The data used to support the findings of this study are available from the corresponding author upon appropriate request.
