# Supplementary material for: Mental health status and related influencing factors of COVID‐19 survivors in Wuhan, China
Source: Clin Transl Med. 2020 Jun 5;10(2):e52. doi: 10.1002/ctm2.52 (PMC7300592; doi:10.1002/ctm2.52)
Supplement: Supplementary file 1 — Supporting information [file CTM2-10-e52-s001.docx]

**Supplementary Table 1**, The Generalized Anxiety Disorder Screener (GAD-7) recording the number of patients who had been bothered by any of the following problems over the last two weeks (N=370).

| Item | Not at all | Several days | More than half the days | Nearly every day |
| --- | --- | --- | --- | --- |
| Feeling nervous, anxious or on edge | 225 (60.8%) | 125 (33.8%) | 8 (2.2%) | 12 (3.2%) |
| Not being able to stop or control worrying | 257 (69.5%) | 92 (24.9%) | 6 (1.6%) | 15 (4.1%) |
| Worrying too much about different things | 292 (78.9%) | 64 (17.3%) | 3 (0.8%) | 11 (3.0%) |
| Trouble relaxing | 312 (84.3%) | 37 (10.0%) | 10 (2.7%) | 11 (3.0%) |
| Being so restless that it is hard to sit still | 333 (90.0%) | 25 (6.8%) | 6 (1.6%) | 6 (1.6%) |
| Becoming easily annoyed or irritable | 319 (86.2%) | 41 (11.1%) | 3 (0.8%) | 7 (1.9%) |
| Feeling afraid as if something awful might happen | 289 (78.1%) | 62 (16.8%) | 10 (2.7%) | 9 (2.4%) |

**Supplementary Table 2**, Patient Health Questionnaire-9 (PHQ-9) recording the number of patients who had been bothered by any of the following problems over the last two weeks (N=370).

|  | Not at all | Several days | More than half the days | Nearly every day |
| --- | --- | --- | --- | --- |
| Little interest or pleasure in doing things | 324 (87.6%) | 36 (9.7%) | 5 (1.4%) | 5 (1.4%) |
| Feeling down, depressed, or hopeless | 314 (84.9%) | 46 (12.4%) | 5 (1.4%) | 5 (1.4%) |
| Trouble falling or staying asleep, or sleeping too much | 261 (70.5%) | 57 (15.4%) | 16 (4.3%) | 36 (9.7%) |
| Feeling tired or having little energy | 281 (75.9%) | 63 (17.0%) | 13 (3.5%) | 13 (3.5%) |
| Poor appetite or overeating | 339 (91.6%) | 23 (6.2%) | 2 (0.5%) | 6 (1.6%) |
| Feeling bad about yourself, or that you are a failure, or have let yourself or your family down | 352 (95.1%) | 14 (3.8%) | 1 (0.3%) | 3 (0.8%) |
| Trouble concentrating on things, such as reading the newspaper or watching television | 339 (91.6%) | 25 (6.8%) | 3 (0.8%) | 3 (0.8%) |
| Moving or speaking so slowly that other people could have noticed? Or the opposite, being so fidgety or restless that you have been moving around a lot more than usual. | 341 (92.2%) | 26 (7.0%) | 1 (0.3%) | 2 (0.5%) |
| Thoughts that you would be better off dead or of hurting yourself in some way | 366 (98.9%) | 4 (1.1%) | 0 (0%) | 0 (0%) |
