# Supplementary figures and images for: Mental health status and related influencing factors of COVID‐19 survivors in Wuhan, China
Source: Clin Transl Med. 2020 Jun 5;10(2):e52. doi: 10.1002/ctm2.52 (PMC7300592; doi:10.1002/ctm2.52)

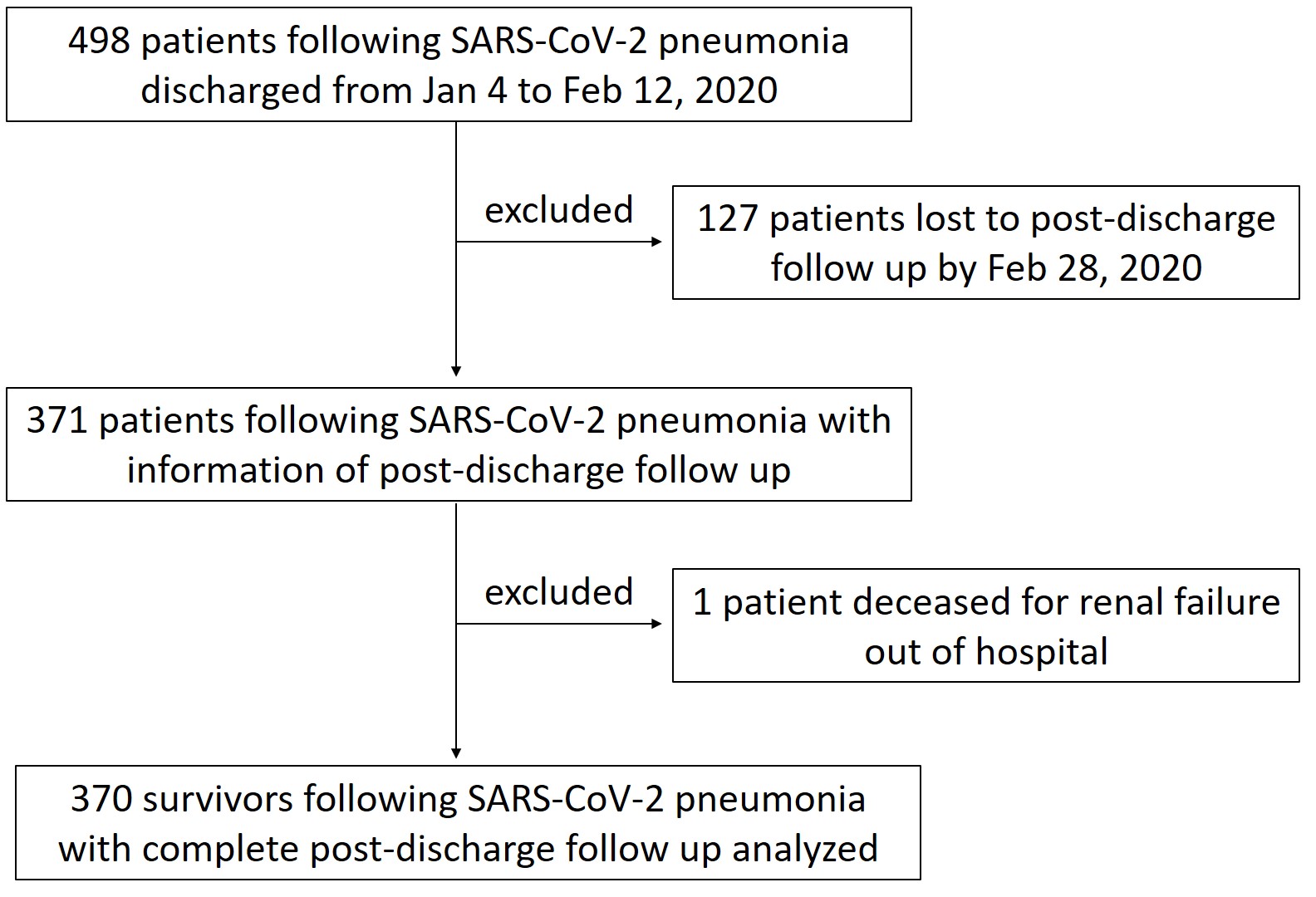

Supplement: Supplementary file 2 — Supporting information [file CTM2-10-e52-s002.jpg]
